# Long Noncoding RNA *OIP5-AS1* Inhibits Cell Apoptosis and Cataract Formation by Blocking *POLG* Expression Under Oxidative Stress

**DOI:** 10.1167/iovs.61.12.3

**Published:** 2020-10-02

**Authors:** Ruihua Jing, Bo Ma, Tiantian Qi, Conghui Hu, Chongbing Liao, Chan Wen, Yongping Shao, Cheng Pei

**Affiliations:** 1 Department of Ophthalmology, The First Affiliated Hospital of Xi'an Jiaotong University, Xi'an, Shaanxi, China; 2 Center for Translational Medicine, Frontier Institute of Science and Technology, Xi'an Jiaotong University, Xi'an, Shaanxi, China

**Keywords:** OIP5-AS1, POLG, apoptosis, oxidative stress

## Abstract

**Purpose:**

Cataract, a clouding of the intraocular lens, is the leading cause of blindness. The lens-expressed long noncoding RNA *OIP5-AS1* was upregulated in lens epithelial cells from patients with cataracts, suggesting its pathogenic role in cataracts. We investigated the regulatory role of *OIP5-AS1* in the development of cataracts as well as potential RNA binding proteins, downstream target genes, and upstream transcription factors.

**Methods:**

Clinical capsules and ex vivo and in vitro cataract models were used to test *OIP5-AS1* expression. Cell apoptosis was detected using Western blots, JC-1 staining, and flow cytometry. Ribonucleoprotein immunoprecipitation-qPCR was performed to confirm the interaction of *OIP5-AS1* and *POLG*. Chromatin immunoprecipitation-qPCR was used to determine the binding of TFAP2A and the *OIP5-AS1* promoter region.

**Results:**

*OIP5-AS1* was upregulated in cataract lenses and B3 cells under oxidative stress. *OIP5-AS1* knockdown protected B3 cells from H_2_O_2_-induced apoptosis and alleviated lens opacity in the ex vivo cataract model. HuR functioned as a scaffold carrying *OIP5-AS1* and *POLG* mRNA and mediated the decay of *POLG* mRNA. *POLG* was downregulated in the cataract lens and oxidative-stressed B3 cells, and *POLG* depletion decreased the mtDNA copy number and MMP, increased reactive oxygen species production, and sensitized B3 cells to oxidative stress-induced apoptosis. *POLG* overexpression reversed these effects. TFAP2A bound the *OIP5-AS1* promoter and contributed to *OIP5-AS1* expression.

**Conclusions:**

We demonstrated that *OIP5-AS1*, activated by TFAP2A*,* contributed to cataract formation by inhibiting *POLG* expression mediated by HuR, thus leading to increased apoptosis of lens epithelial cells and aggravated lens opacity, suggesting that *OIP5-AS1* is a potential target for cataract treatment.

Cataract is a clouding of the natural intraocular crystalline lens that leads to blindness, and it represents the most common eye disease globally, with age-related cataract, the most common form. The main causes of ARCs include excessive apoptosis of human lens epithelial cells (HLECs), reactive oxygen species (ROS) production, and dysfunction of the α-crystalline chaperone.^1^^,^^2^ The main ROS in the eye are superoxide anion (O_2_^−^), H_2_O_2_ and hydroxyl radical (OH^−^), which cause oxidative damage to DNA and proteins in the lens and lead to cell apoptosis.^3^^,^^4^ Excessive apoptosis of lens epithelial cells is the early common event of age-related cataract. Previous studies discovered that caspase 1-dependent pyroptosis of HLECs contributes to cataract formation^5^.^5^ Despite these findings, the molecular mechanisms underlying the pathogenesis of cataracts are still unclear.

Noncoding RNA (ncRNA) are functional molecules that regulate gene expression or modification through different mechanisms. Both short and long types of ncRNA could modulate gene expression and epigenetic processes, including methylation, histone modification, chromatin remodeling, and gene silencing^6^.^6^ Long ncRNAs (lncRNAs) are a class of noncoding transcripts longer than 200 nucleotides that play various regulatory roles in a wide array of cellular processes^7^.^7^ Many lncRNAs have been identified in the lens, and some have been shown to participate in the regulation of HLEC activities.^8^^,^^9^ For instance, *MIAT* acts as a competing endogenous RNA to form a feedback loop with Akt and miR-150-5p, thus regulating HLEC apoptosis and migration.^10^ *MALAT1* can promote the apoptosis and oxidative stress of HLECs through the activation of p38 MAPK^11^.^11^ However, the majority of lens-expressed lncRNAs have not been functionally characterized.

The lncRNA *OIP5-AS1* (known as *Cyrano* in zebrafish) is highly and ubiquitously expressed in various tissues and species^12^.^12^ *Cyrano* has been shown to promoting zebrafish brain development, and silencing of *Cyrano* by miR-7 without changing zygotic *Cyrano* altered the brain architecture at 24 hours post fertilization and 48 hours post fertilization^13^.^13^ In the lens, *OIP5-AS1* is upregulated in the HLECs of patients with cataracts.^10^ This molecule has been reported as a tumor suppressor or oncogene depending on the tumor type.^14^^,^^15^ *OIP5-AS1* is also involved in the self-renewal of embryonic stem cells by modulating the expression of miR-7.^12^^,^^16^ In human osteosarcoma U2OS cells, *OIP5-AS1* depletion increases the expression of *POLG*, a mitochondrial DNA polymerase responsible for the synthesis of mitochondrial DNA (mtDNA), and RNA pulldown studies showed that *OIP5-AS1* is associated with *POLG* mRNA.^17^ Mutations of *POLG* were reported to contribute to the formation of cataracts and progressive external ophthalmoplegia.^18^^,^^19^ However, whether *OIP5-AS1* affects cataract formation by influencing *POLG* expression and the underlying regulatory mechanism have not been clarified.

In this study, we investigated the role of *OIP5-AS1* in the development of cataracts in vitro and ex vivo and whether it interferes with *POLG* expression. We also attempted to identify the upstream regulatory factors of *OIP5-AS1* in B3 cells.

## Methods

### Culture of the HLEC Line B3

The HLEC line B3 (CRL-11421, American Type Culture Collection, Manassas, VA) was used for all of the cell experiments. Cells were cultured in DMEM (CM10014, Macgene) supplemented with 10% fetal bovine serum (04-001-1ACS, Biological Industries, Cromwell, CT) and 1% penicillin-streptomycin (SV30010, HyClone, Logan, UT) at 37°C in a humidified atmosphere with 5% CO_2_. H_2_O_2_ was applied to induce oxidative stress in the medium. A total of 10^6^ cells within 12 passages were seeded in culture flasks, and the medium was renewed every 2 days. All cells were free of mycoplasma.

### Clinical Sample Collection

This study was approved by the Ethics Committee of the First Affiliated Hospital of Xi'an Jiaotong University and performed according to the Declaration of Helsinki. All of the lens capsules from the patients with cataracts were obtained at Xi'an Jiaotong University during cataract surgery. All of the patients provided informed consent, and the nature and possible consequences of the study were explained. All of the control lenses were from the Eye Bank of the First Affiliated Hospital of Xi'an Jiaotong University, and the capsules were acquired by continuous curvilinear capsulorhexis. All of the samples were preserved in TRIzol. Detailed information on the clinical participants is provided in Supplementary Table S1.

### Establishment of the Rat Model of Cataract

Twenty healthy adult SD rats (provided by the Animal Experimentation Center Affiliated with Xi'an Jiaotong University, which adhered to the ARVO Statement for the Use of Animals in Ophthalmic and Vision Research) weighing 180 to 200 g with a gender ratio of 1:1 were chosen for this study. According to Lim,^20^ UVB is widely used to induce cataracts in rats. All rats were exposed to 9 kJ/m^2^ at 302 nm UVB (UVLM-26, UVP) within 7 days and then sacrificed 3 days after exposure. All of the rats were mydriatic before being anaesthetized by pentobarbital sodium, and all of the left eyes served as controls. A large incision was made using a 2.0-mm paracentesis knife, and then the intact lenses were isolated along the limbus and placed in warm PBS. Next, the zonular region was carefully cut away. Images were taken by a Canon M6 system.

### Nuclear and Cytoplasmic OIP5-AS1 mRNA Quantification

Cell collection and fractionation were performed according as described by Wang et al^21^.^21^ B3 cells (approximately 3 × 10^6^ cells) were digested using trypsin and collected in cold PBS. Then, the cells were centrifuged and resuspended in RSB buffer and lysed in RSBG40. The supernatant from high-speed centrifugation was collected as the cytoplasmic fraction, and the precipitated nuclear substances were released in RSBG40 containing one-tenth volume of detergent (3.3% [wt/wt] sodium deoxycholate and 6.6% [vol/vol] Tween-40). RNA was extracted using TRIzol, and RT-qPCR was used for quantification. Whole-cell RNA was used for normalization. The primers used are listed in Table 1.

**Table 1.** Primers Used for RT-qPCR

Gene	Forward	Reverse
h-OIP5-AS1	TTTCGTGGATGCCACAGGAC	TAGTTCCTCTCCTCTGGCCG
h-GAPDH	GTCAAGGCTGAGAACGGGAA	AAATGAGCCCCAGCCTTCTC
h-ACTIN	TACCTCATGAAGATCCTCACC	TTTCGTGGATGCCACAGGAC
h-POLG	TAGTTCCTCTCCTCTGGCCG	GAGGCAGCTTGAAAAACCAG
h-HuR	GTCATGATGGCGGAGTAAT	AGGACCCGCGAGTTGATGATCCG
h-7SL	ACTGAGCAGTCCACGTAGAAA	GGCTGGAGTGCAGTGGCTAT
h-MALAT1	GATTGAGGAGGCTGTGCTGT	CAGCTGCCTGCTGTTTTCTG
r-OIP5-AS1	AGGACCCGCGAGTTGATGATCCG	ACTGAGCAGTCCACGTAGAAA
r-ACTIN	CCATGCTCGTTCAGTGACTACCTC	AAGGGTGTAAAACGCAGCTC
h-TFAP2A	GTTCACGCCGATCCATGAAAA	AGATTGACCTACAGTGCCCAG
mt-ACTIN	ATCATGTTTGAGACCTTCAAC	ATCTCTTGCTCGAAGTCCA
mt-ND1	TCTAGCCACCTCTAGCCTAG	GTCATGATGGCGGAG TAAT
ChIP-region1	GGAAACATTCATCGAGGGGC	AACCAGAGTATGTCACCGCT
ChIP-region2	AGAATCCACCCAGATGACAGC	GCAGGGAGGGTATACGTGGG
ChIP-region3	GGGCCTTCAGAAAAGTGACG	GCGCCCAGGCTTTTAATCCA
ChIP-region4	CCTCGGGCCTGAGAATGAAA	TTTCGATCCGGATTGGGTCC
ChIP-region3’	ATGCAGAAGAAAGGATGCCCT	CCCTCCTGCATAGAGGAAACC

### Western Blot

Whole cells were lysed using Laemmli sample buffer (1610737, Bio‑Rad, Hercules, CA). Proteins were separated by SDS-PAGE and transferred to a polyvinylidene difluoride membrane. For Western blot analysis, the membrane was blocked in 1% BSA for 1 hour and then incubated in primary antibodies that recognize POLG (ab128899, Abcam, Cambridge, UK), Bax (ab32503, Abcam), Bcl-2 (ab32124, Abcam), and caspase3 (#9664, CST). After incubation with appropriate secondary antibodies, the protein signals were detected using an enhanced chemiluminescence Western blotting detection kit (1705060, Bio-Rad).

### Small Interfering RNA (siRNA) Knockdown and Plasmid Transfection

Transfection of siRNA was performed as described by the data sheet. Briefly, 30 pM of siRNA and 9 µL of RNAiMAX reagent were diluted in Opti-MEM and then the diluted siRNA was added to the diluted lipofectamine RNAiMAX Reagent (1:1 ratio). After 5 minutes of incubation at room temperature, the siRNA-lipid complex was added to the cells. The sequences were as follows: siRNA for human *HuR*: TTACCAGTTTCAATGGTCA; siRNA for human *OIP5-AS1*: CACCAAACAGGCUUUGUGUUCCUTA; siRNA for human *POLG*: 1: GGUGCACAGACUUUAUGUA, 2: GGAUGGUAAUAGCUGUAAUTT; siRNA for rat *OIP5-AS1*: 1: GGTTTGTTACAGTAGTGAA, 2: GGTTAGTCAGATTGGACAA, 3: GGAAGTACAAGATAAACAA; siRNA for *TFAP2A*: AACAUCCCAGAUCAAACUGUA.

All sequences were from Guangzhou RiboBio Co., Ltd. Two POLG siRNAs were mixed and transfected into cells together. The POLG overexpression plasmid was purchased from Shanghai Genechem Co., Ltd (Shanghai, China). The *POLG* plasmid or negative control vector was transfected using jetPRIME (Polyplus) according to the manufacturer's instructions. Briefly, 2 µg of vector and 4 µL of jetPRIME transfection reagent were added to 200 µL of jetPRIME buffer and incubated for 15 minutes at room temperature. Then, the mix was added to the medium.

### Mitochondrial Membrane Potential (MMP) Assay With JC-1

After appropriate treatment, the cells were washed in PBS twice, and 2 µM JC-1 (C2006, Beyotime, Shanghai, China) was loaded. Then, the cells were incubated at 37°C for 20 minutes in the dark. Cell visualization was performed by a fluorescence microscope (Eclipse Ti, Tokyo, Japan), and the red/green fluorescence intensity was determined by a microplate reader (SpectraMax i3, Molecular Devices, Eugene, OR).

### Quantitative RT-qPCR

Total RNA samples from the rat lens or cultured cells were extracted using TRIzol (Invitrogen, Carlsbad, CA) according to the manufacturer's instructions. The iScript gDNA Clear cDNA Synthesis Kit (1725035, Bio-Rad) was used for cDNA synthesis, and SsoFast EvaGreen (1725201AP, Bio-Rad) was used for real-time PCR. The primers are listed in Table 1.

### Analysis of mtDNA Copy Number

Whole-cell DNA was extracted using an assay kit from Tiangen Biotech Co., Ltd. (Beijing, China), and 50 ng DNA was used as the template for RT-qPCR. Actin was used as a nuclear single copy control and analyzed as described by Foote et al.^22^ The primers used in this assay are listed in Table 1.

### ROS Detection

After induction of H_2_O_2_ for 24 hours, 10 µM DCFH-DA (D6883, Sigma-Aldrich, St Louis, MO) was added to the medium and the cells were incubated at 37°C for 30 minutes in the dark. Then, the cells were washed with PBS and resuspended. The ROS mean intensity was measured using a CytoFLEX system (Beckman Coulter, Brea, CA) in the FITC channel. The excitation wavelength was 488 nm, and the emission wavelength was 525 nm.

### Ribonucleoprotein Immunoprecipitation (RIP)-qPCR

Ribonucleoprotein is composed of RNA and RNA-binding proteins and can regulate post-transcriptional gene expression. For the RIP assay, Protein A/G PLUS-Agarose beads (sc-2003, Santa Cruz Biotechnology, Santa Cruz, CA) were precoated with 5 µg HuR antibody (ab200342, Abcam) or rabbit normal IgG overnight at 4°C. A total of 10^7^ cells were prepared in NT-2 buffer with 1 mM DTT and recombinant RNasin ribonuclease inhibitor. Then, the lysates were incubated with precoated agarose beads overnight at 4°C. After six washes in NT-2 buffer, RNA was extracted using TRIzol. RT-qPCR was used to detect enrichment. Primers for *OIP5-AS1* and *POLG* are shown in Table 1.

### Establishment of a Rat Ex Vivo Model of Cataract

Rat selection and animal care were performed as described elsewhere in this article. For the analysis of ex vivo opacified lens, the intact lenses were isolated along the limbus and the zonular region was carefully cut away. Whole lenses were cultured in DMEM containing 5% fetal bovine serum and 1% penicillin-streptomycin. Sixty picomoles of siRNA were supplied to silence rat *OIP5-AS1*. The lenses were visualized using a Canon M6 system.

### Annexin V/PI Apoptosis Assay

Cell apoptosis was detected using an APC-PE-PI Apoptosis Assay kit (Biolegend, San Diego, CA) on a CytoFLEX system. All of the cells in the plate along with floating cells after treatment with 200 µM H_2_O_2_ for 24 hours were collected and washed in PBS. Then, the cells were resuspended in 100 µL of annexin V binding buffer. Next, 5 µL of annexin V together with 10 µL of propidium iodide was added to the suspension. After 15 minutes of incubation in the dark at room temperature, 400 µL of binding buffer was added to terminate the reaction and the cells were counted by flow cytometry.

### Motif Prediction

We first selected the differentially expressed genes between control and patient lens epithelial cells from Wu et al^23^ and prioritized genes upregulated in HLECs from the cataract group. Then, motif prediction on *OIP5-AS1* was performed using FIMO from the MEME Suite toolkit (v4.11.0)^24^ with default parameters and motifs available from five public motif databases: JASPAR (2018 version)^25^,^25^ HOCOMOCO (v11)^26^,^26^ SwissRegulon,^27^ Transfac, and Jolma2013^28^.^28^ We selected the *OIP5-AS1* promoter sequence (–2000 bp to +200 bp) for the motif analysis.

### Chromatin Immunoprecipitation (ChIP)-qPCR

ChIP assays were performed for the B3 cells. All of the B3 cells in the culture dish (approximately 1 × 10^7^ cells) were first crosslinked using methanol and quenched by glycine. Then, the cells were rinsed and scraped off into conical tubes. The pellets were resuspended in cell lysis buffer and SDS lysis buffer in turn, and a VirTis Virsonic 100 Ultrasonic Homogenizer/Sonicate (VCX500, Socics, Connecticut, USA) was used to shear chromatin. The sonicated lysates were diluted using DNA dilution buffer and precleared with protein A/G PLUS-agarose beads for 2 hours. Two percent of the total supernatant was taken as the input. Two micrograms of rabbit normal IgG or TFAP2A antibody (ab52222, Abcam) and 40 µL of beads were added together for immunoprecipitation. The DNA–protein complex was eluted, reverse crosslinked, and purified for RT-PCR. The PCR primers were designed according to the four clusters to evaluate the IP efficacy of the four regions as shown in Table 1. Because ultrasound can break the chromatin fragments into independent nucleosomes, the 3' primer pair was used to amplify short regions (<180 bp) in region 3 for ChIP-qPCR.

### Statistical Analysis

GraphPad Prism 6.0 statistical software was applied to perform the statistical analyses of the data. All of the data are presented as the mean ± SEM from at least three independent experiments. The differences between two groups were analyzed by unpaired Student *t* tests, comparisons between multiple groups were analyzed using one-way ANOVAs, and a *P* value of less than 0.05 was considered significant. The grade count data of the isolated turbid lens in Table 2 were analyzed using the Kruskal–Wallis test and the Mann–Whitney post hoc test with a Bonferroni correction in SPSS 21.0 (IBM Corp., Armonk, NY), with a *P* value of less than 0.05/6 considered statistically significant.

**Table 2.** Morphologic Assessment of Lenticular Opacification of Lenses After 6 Days Incubation

	Grade 0	Grade I	Grade II	Grade III	Grade IV
siRNA-NC	2	3	0	0	0
siOIP5-AS1	2	3	0	0	0
siRNA-NC+H_2_O_2_	0	0	0	0	5
siOIP5-AS1+H_2_O_2_	0	1	3	1	0

Incidence and grading of cataract formation of the lenses in each group. Grade 0: Absence of opacification (grid lines clearly visible); grade 1, minimal clouding of lens (grid lines easily visible); grade 2, presence of flaky opacification involving the part of lens (with gridlines visible), grade 3, Presence of opacification of the entire of lens (with grid lines faintly visible), and grade 4, presence of extensive opacification of the entire lens (grid lines not visible).

## Results

### *OIP5-AS1* Was Upregulated in Clinical Cataract Samples and Cataract Models

**Figure 1.**
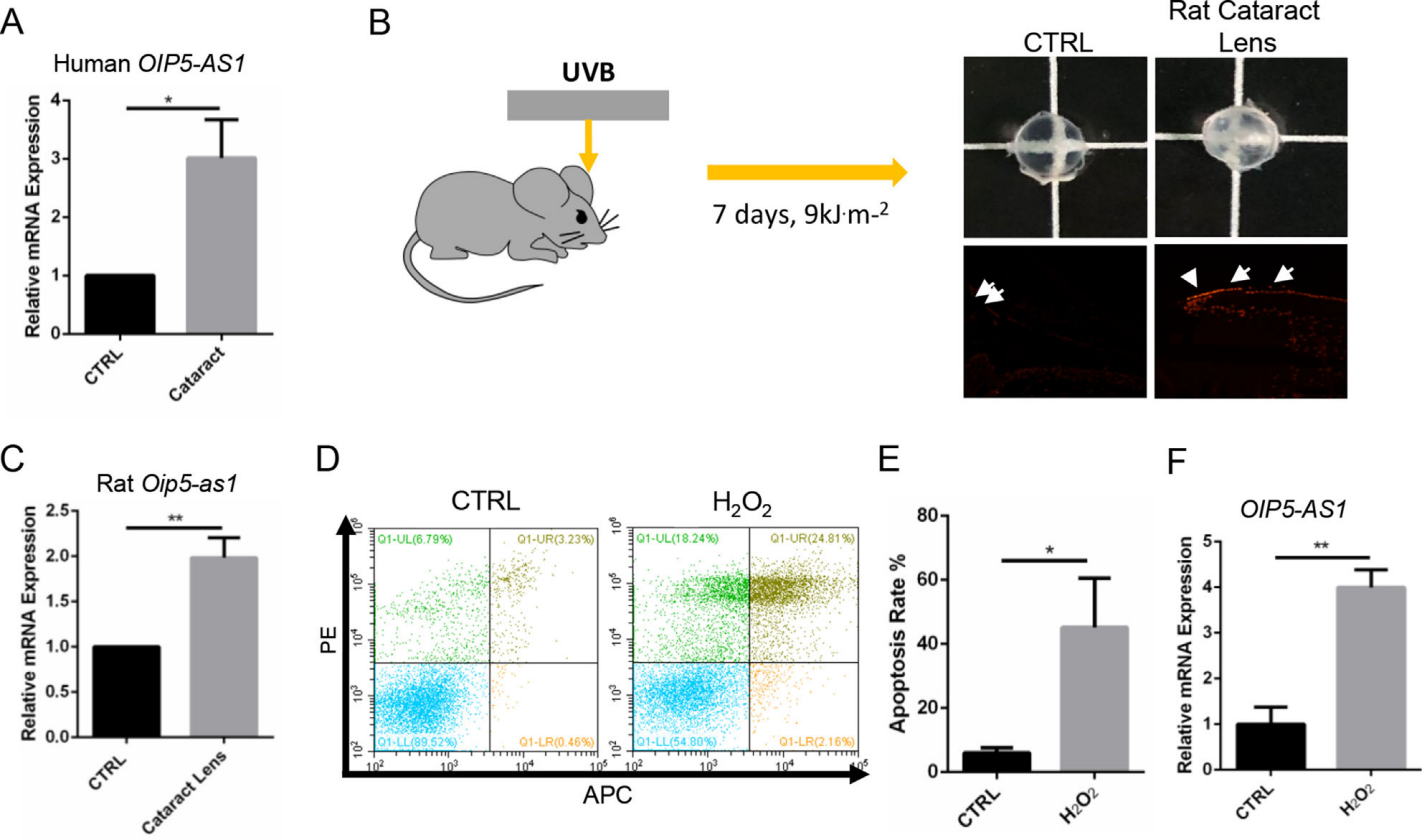
*OIP5-AS1* expression was upregulated in clinical lens samples and cataract models. (**A**) RT-qPCR of *OIP5-AS1* expression in lens capsules. (**B**) Rats underwent a total of 9 kJ.m^2^ UVB irradiation for 7 days and were then sacrificed. Their lenses were isolated, photographed, and stained using TUNEL dye for observation. The *white arrows* indicate the TUNEL-positive cells. (**C**) *Oip5-as1* was upregulated in the lens of rats with cataract as shown by RT-qPCR. (**D–E**) H_2_O_2_ (200 µM) was chosen for the oxidative stress cell model. (**F**) *OIP5-AS1* increased in the oxidative stress cell model, as shown by RT-qPCR. *P < 0.05, **P < 0.01.

We first tested *OIP5-AS1* expression in the clinical samples. RT-qPCR showed that the expression of *OIP5-AS1* in the lens epithelial cells from the cataract group was much higher than that in the control group (Fig. 1A, *P* < 0.05). We then investigated the expression of *Oip5-as1* in an in vivo rat model of UVB-induced cataract. Compared with the control lens, the UVB-radiated lens became turbid after 7 days and displayed an impaired tissue structure (Supplementary Fig. S1A). The opacification of the UVB-irradiated lens was associated with an increased apoptosis rate as shown by enhanced TUNEL staining (Fig. 1B) and increased expression of *Oip5-as1* (*P* < 0.01, Fig. 1C), cleaved caspase 3 and Bax (Supplementary Fig. S1B). Moreover, we tested the expression of *OIP5-AS1* in an H_2_O_2_-induced B3 cell model. The H_2_O_2_ treatment increased cell apoptosis from approximately 6% to 42% (*P* < 0.05, Figs. 1D, 1E) and concomitantly decreased the mtDNA copy number of B3 cells (*P* < 0.05, Supplementary Fig. S1C). Notably, *OIP5-AS1* expression increased in response to the H_2_O_2_ treatment (*P* < 0.01, Fig. 1F). These results showed that *OIP5*-*AS1* was upregulated in the lens epithelial cells in cataracts. Additionally, in ex vivo and in vitro cataract models, oxidative stress induced *OIP5-AS1* expression.

### *OIP5-AS1* Knockdown Alleviated ROS Production and Cell Apoptosis

**Figure 2.**
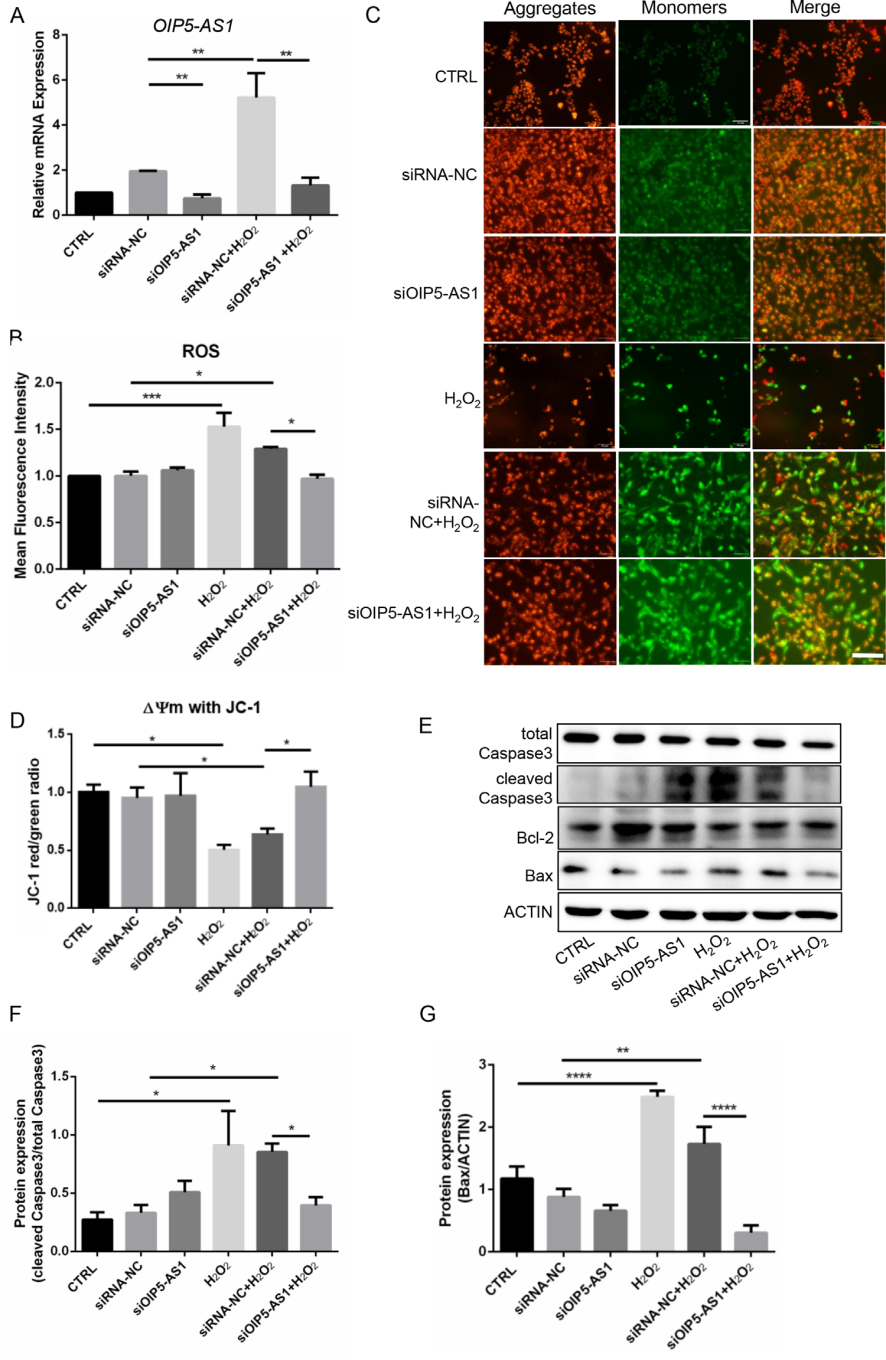
*OIP5-AS1* knockdown alleviated cell apoptosis and ROS production. (**A**) mRNA level of *OIP5-AS1* quantified by RT-qPCR. SiRNA silencing abolished H_2_O_2_-enhanced OIP5-AS1 expression. (**B**) Flow cytometric detection of the mean fluorescence intensity of the cell ROS. (**C–D**) Analysis of MMP staining by JC-1with a fluorescence microscope and microplate spectrophotometer. The JC-1 monomer was *green*, indicating a low MMP, and JC aggregates were *red*, indicating a high MMP. Scale bar: 500 µm. (**E**) Western blot of apoptosis-associated proteins. (**F**–**G**) Quantification of the Western blot results. *P < 0.05, **P < 0.01, ***P < 0.0001, ****P < 0.00001.

Oxidative stress-induced apoptosis of HLECs is one of the major contributing factors to cataract formation.^4^ To investigate the potential regulatory role of *OIP5-AS1* in oxidative stress-induced apoptosis, we silenced *OIP5-AS1* in the H_2_O_2_-treated B3 cells. H_2_O_2_ dramatically induced *OIP5-AS1* upregulation, whereas siRNA silencing abolished the H_2_O_2_-enhanced *OIP5-AS1* expression (*P* < 0.01, Fig. 2A). Under oxidative stress, the *OIP5-AS1* knockdown group produced less ROS (*P* < 0.05, Fig. 2B) than the negative control group. We conducted JC-1 staining to measure MMP to demonstrate the early stage of cell death. As shown by fluorescence microscopy, H_2_O_2_ significantly reduced the MMP of B3 cells, but knockdown of *OIP5-AS1* effectively blocked the decrease in MMP under oxidative stress (*P* < 0.05, Figs. 2C, 2D). Apoptosis is a common mechanism of generating cataracts.^1^ We further detected mitochondrial apoptosis-associated proteins, such as Bcl-2, Bax and cleaved caspase 3. The H_2_O_2_ treatment increased cleaved caspase 3 and Bax protein expression, although *OIP5-AS1* knockdown decreased the levels under oxidative stress, but did not increase the protein expression (Figs. 2E-2G). These results confirmed that *OIP5-AS1* contributed to oxidative stress-induced ROS production and the mitochondrial apoptosis pathway in B3 cells.

### *OIP5-AS1* Knockdown Prevented Lens Opacity Ex Vivo

**Figure 3.**
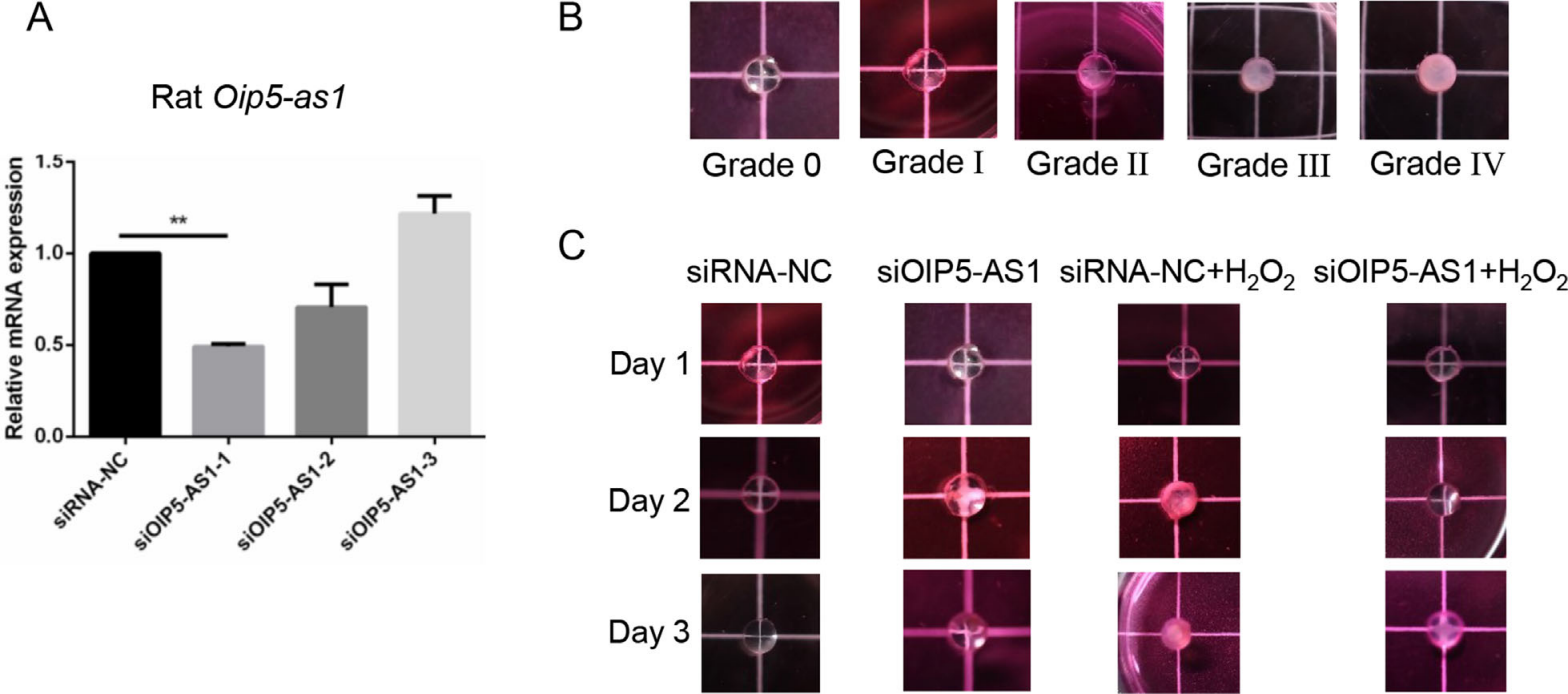
*OIP5-AS1* knockdown might be effective in preventing lens opacity ex vivo. (**A**) *OIP5-AS1* knockdown efficiency of siRNA in the rat lens. (**B**) Grades of isolated lenses on a scale of 0 to 4. (**C**) The lenses were kept in DMEM and photographed by a Canon M6 system. **P < 0.01.

*OIP5-AS1* is highly abundant in humans, rats, zebrafish, and chickens, indicating its importance. To verify the effects of *OIP5-AS1*, we established an ex vivo cataract model as described by Xiang et al 2018.^29^ After analyzing the efficacy of various siRNAs, an effective siRNA (siOIP5-AS1-1) (*P* < 0.01, Fig. 3A) was used in the following experiments. The clouding of the lens was evaluated by a doctor blinded to the study groups based on grade assessment^8^^,^^30^ on a scale of 0 to 4 as shown in Figure 3B. The counts of lens stages in every group are shown in Table 2. We observed that *Oip5-as1* knockdown did not influence the opacity of the lens compared with the that of the negative control siRNA group (*P* > 0.008, Fig. 3C). With the H_2_O_2_ treatment, the lens in the negative siRNA transfection group became more turbid (*P* < 0.008, Fig. 3C) than that in the *Oip5-as1* knockdown group, indicating the success of our H_2_O_2_-induced ex vivo cataract model. Notably, under oxidative stress, the lens in the *Oip5-as1* silencing group was not turbid compared with that in the negative siRNA transfection group (*P* < 0.008, Fig. 3C). In conclusion, *Oip5-as1* knockdown might prevent lens opacity under oxidative stress. *OIP5-AS1* could be a new target for the treatment of cataracts.

### *POLG* Was Negatively Regulated by *OIP5-AS1* With HuR

**Figure 4.**
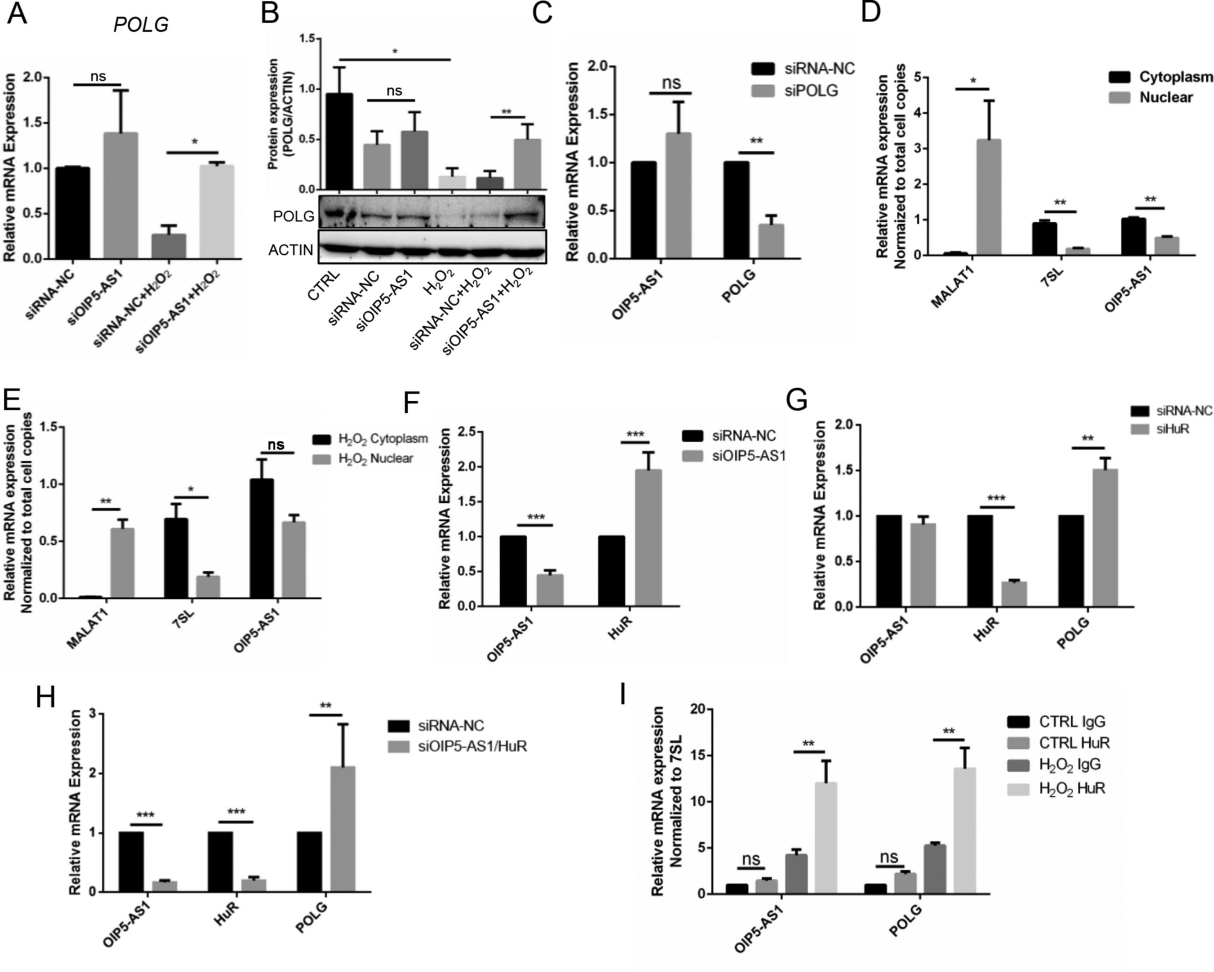
HuR bound *OIP5-AS1* and *POLG* mRNA and mediated the decay of *POLG* mRNA. (**A**) *POLG* mRNA expression after *OIP5-AS1* knockdown with or without H_2_O_2_ shown by RT-qPCR. (**B**) POLG protein expression after *OIP5-AS1* knockdown with or without H_2_O_2_ shown by Western blots and quantification with ImageJ. (**C**) RT-qPCR detection of *OIP5-AS1* expression after *POLG* knockdown. (**D**) RT-qPCR determination of *OIP5-AS1* cell distribution in fractions of B3 cells. *MALAT1* localized in the nucleus, and *7SL* was expressed in the cytoplasm. (**E**) RT-qPCR determination of *OIP5-AS1* cell distribution in cell fractions of B3 cells treated with H_2_O_2_. *MALAT1* localized in the nucleus, and *7SL* was expressed in the cytoplasm. (**F**) *HuR* mRNA expression after *OIP5-AS1* knockdown shown by RT-qPCR. (**G**) *OIP5-AS1* and *POLG* mRNA expression after *HuR* silencing shown by RT-qPCR. (**H**) *POLG* mRNA expression was upregulated after both *OIP5-AS1* and *HuR* knockdown. (**I**) RIP detection of HuR binding of the mRNA of *OIP5-AS1* and *POLG* with or without H_2_O_2_ stress. *P < 0.05, **P < 0.01, ***P < 0.001, ns: *P* > 0.05.

Previous research has suggested that *OIP5-AS1* could negatively regulate *POLG* in the U2OS cell line^17^.^17^ Consistently, knockdown of *OIP5-AS1* significantly increased *POLG* expression at both the mRNA and protein levels in the H_2_O_2_-induced B3 cells (*P* < 0.05, *P* < 0.01, Figs. 4A–4B). Additionally, we did not observe a significant change in the *OIP5-AS1* expression after *POLG* knockdown, indicating that *OIP5-AS1* was the upstream regulator of *POLG* (Fig. 4C). We further performed mRNA quantification on the cell fractions of the *OIP5-AS1* cells. Compared with *MALAT1* (in the nucleus) and *7SL* (in the cytoplasm), *OIP5-AS1* was found more frequently in the cytoplasm than the nucleus in both normal and H_2_O_2_-induced B3 cells (Figs. 4D, 4E). Generally, diverse lncRNA functions require interactions with one or more RNA binding proteins,^31^ thus inspiring our investigation of potential key RNA binding proteins in mediating the *OIP5-AS1-POLG* interaction. Previous research showed that *OIP5-AS1* interacted with HuR at 4 sites.^32^ HuR has 3 target sites on *OIP5-AS1* and 39 target sites on *POLG* in starBase (http://starbase.sysu.edu.cn/starbase2/browseRbpLncRNA.php), suggesting the important role of HuR in the *OIP5-AS1-POLG* interaction. In our study, *OIP5-AS1* knockdown increased HuR mRNA expression (*P* < 0.001, Fig. 4F). *HuR* knockdown did not change the expression of *OIP5-AS1*, but upregulated *POLG* mRNA (*P* < 0.01, Fig. 4G). Simultaneous *OIP5-AS1* and *HuR* knockdown enhanced *POLG* mRNA expression (*P* < 0.01, Fig. 4H). Although HuR has a well-known role in mRNA stabilization,^33^ this molecule was also found to destabilize p16 mRNA together with AUF1,^34^ which was consistent with our finding that HuR could suppress *POLG* expression. To further demonstrate the binding of HuR to both *OIP5-AS1* and *POLG* mRNA in vitro, we performed RIP assays of B3 cells. The results showed that the specific HuR antibody bound to both *OIP5-AS1* mRNA and *POLG* mRNA in normal HLECs at low affinity. However, we observed apparently higher binding of HuR on *OIP5-AS1* mRNA and *POLG* mRNA than that of the IgG group in B3 cells under oxidative pressure (*P* < 0.01, Fig. 4I). Together, these results suggested that HuR might function as a scaffold carrying *OIP5-AS1* and *POLG* mRNA and mediate the decay of *POLG* mRNA when lens oxidative stress is excessive.

### Depletion of *POLG* Increased B3 Cell Apoptosis

**Figure 5.**
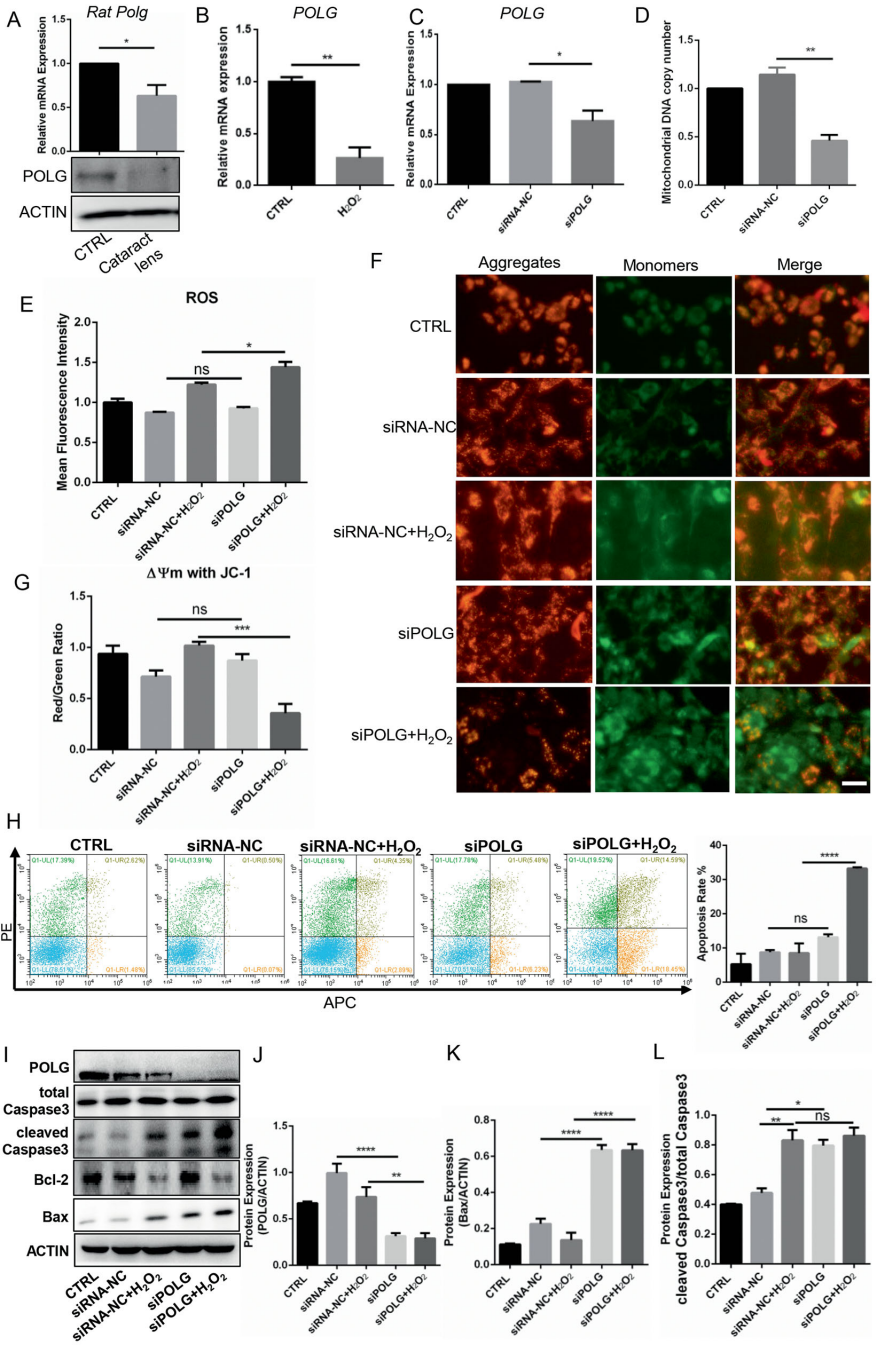
*POLG* knockdown increased the sensitivity of B3 cells to ROS. (**A**) Detection of *Polg* mRNA expression in the lenses of rats with cataracts by RT-qPCR. (**B**) *POLG* mRNA expression in the oxidative stress cell model shown by RT-qPCR. (**C**) Efficacy of *POLG* siRNA knockdown shown by RT-qPCR. (**D**) MtDNA copy number after *POLG* silencing shown by RT-qPCR. (**E**) Flow cytometric detection of the mean fluorescence intensity of ROS. (**F**–**G**) Microplate spectrophotometer analysis of MMP by JC-1 staining. The JC-1 monomer was *green*, representing a low MMP, and JC aggregates were *red*, representing a high MMP. (**H**) Flow cytometry was used to test apoptosis of the *POLG*-silenced cells with or without H_2_O_2_ loading and apoptotic cell rate quantification. (**I**) Western blot characterization of apoptosis-associated protein and POLG expression. (**J**–**L**) Quantification of the Western blot results. Scale bar: 100 µm. *P < 0.05, **P < 0.01, ***P < 0.0001, ****P < 0.00001, ns: *P* > 0.05.

To explore the precise mechanism of *POLG* in cataracts, we first measured *POLG* expression in rat and cell models and detected significantly decreased *POLG* expression in both lens samples from a rat cataract model (*P* < 0.05, Fig. 5A) and a cell model (*P* < 0.01, Fig. 5B). We further knocked down *POLG* expression in B3 cells (*P* < 0.05, Fig. 5C) and detected a significant reduction in mtDNA copy number (*P* < 0.01, Fig. 5D), indicating the important role of *PLOG* in mitochondrial function. The mitochondrial electron transfer chain was reported to be one of the sources of ROS, suggesting the potential role of *POLG* in ROS. Indeed, we observed that *POLG* depletion increased the ROS level (*P* < 0.05, Fig. 5E) under oxidative stress. We also observed a decrease in MMP after *POLG* depletion under oxidative stress, indicating early apoptosis (*P* < 0.01, Figs. 5F, 5G). Finally, *POLG* knockdown contributed to an increase in both apoptotic cells (Fig. 5H) and proapoptotic protein expression (Bax and cleaved caspase 3) (Figs. 5I, 5L). Moreover, when H_2_O_2_ was added, *POLG* knockdown further increased the number of apoptotic cells (Fig. 5H) and Bax protein expression (Figs. 5I, 5K). Together, these results indicated the role of *PLOG* in mtDNA replication, ROS production and cell apoptosis, indicating that *POLG* depletion in B3 cells increased ROS production and induced cell apoptosis under oxidative stress.

### Overexpression of *POLG* Maintained B3 Cell Viability

**Figure 6.**
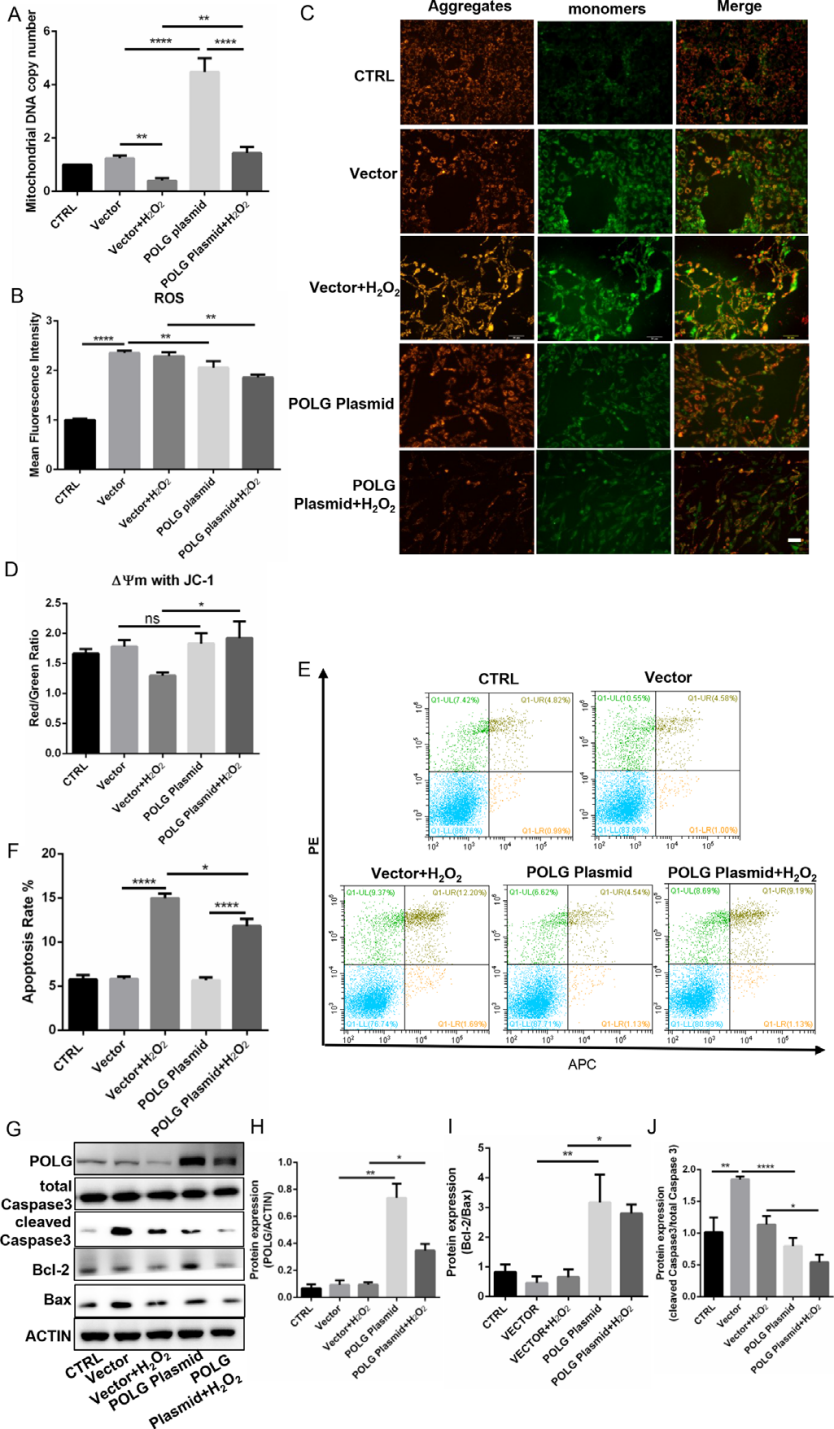
*POLG* maintained cell viability. (**A**) MtDNA copy number examination by RT-qPCR. (**B**) Flow cytometric detection of the mean fluorescence intensity of ROS in the cells transfected with the null vector or the POLG plasmid under oxidative stress. (**C**, **D**) Fluorescence microscopy and microplate spectrophotometry analysis of MMP by JC-1 staining. The JC-1 monomer was *green*, representing a low MMP, and JC aggregates were *red*, representing a high MMP. (**E**) Cell apoptosis detected by flow cytometry. (**F**) Apoptosis rate quantification of E. (**G**) Western blot analysis of apoptosis-associated proteins and *POLG* expression. (**H**–**J**) Quantification of the Western blot results. Scale bar: 100 µM. *P < 0.05, **P < 0.01, ****P < 0.00001, ns: *P* > 0.05.

We demonstrated that *POLG* silencing could promote ROS production and cell apoptosis. Then, the following experiments were conducted to confirm whether *POLG* overexpression could reverse these effects. Our results showed that the mtDNA copy number was higher in the *POLG* vector transfection group than the null-vector group in B3 cells treated with H_2_O_2_ (*P* < 0.01, Fig. 6A). Moreover, transfection of the *POLG* overexpression vector decreased ROS generation compared to that of the control group in the H_2_O_2_-induced B3 cells (*P* < 0.05, Fig. 6B), although vector transfection caused excessive ROS production (fold change > 2, *P* < 0.0001, Fig. 6B). With H_2_O_2_ treatment, *POLG* overexpression rescued the decrease in MMP observed in the JC-1 analysis (*P* < 0.05, Figs. 6C, 6D) and the cell apoptosis of B3 cells detected by flow cytometry (*P* < 0.05, Figs. 6E, 6F). Finally, we detected significantly decreased expression of two proapoptotic proteins (cleaved caspase 3 and bax) in the *POLG* overexpression groups compared with the null-vector group (Figs. 6G–6J) under oxidative stress. Thus, *POLG* overexpression could maintain cell viability, avoid excessive ROS production, restrain mtDNA depletion and suppress apoptotic pathway activation, indicating that *POLG* was responsible for ROS production and cell survival under oxidative stress.

### TFAP2A Was Responsible for *OIP5-AS1* Transcriptional Activation

**Figure 7.**
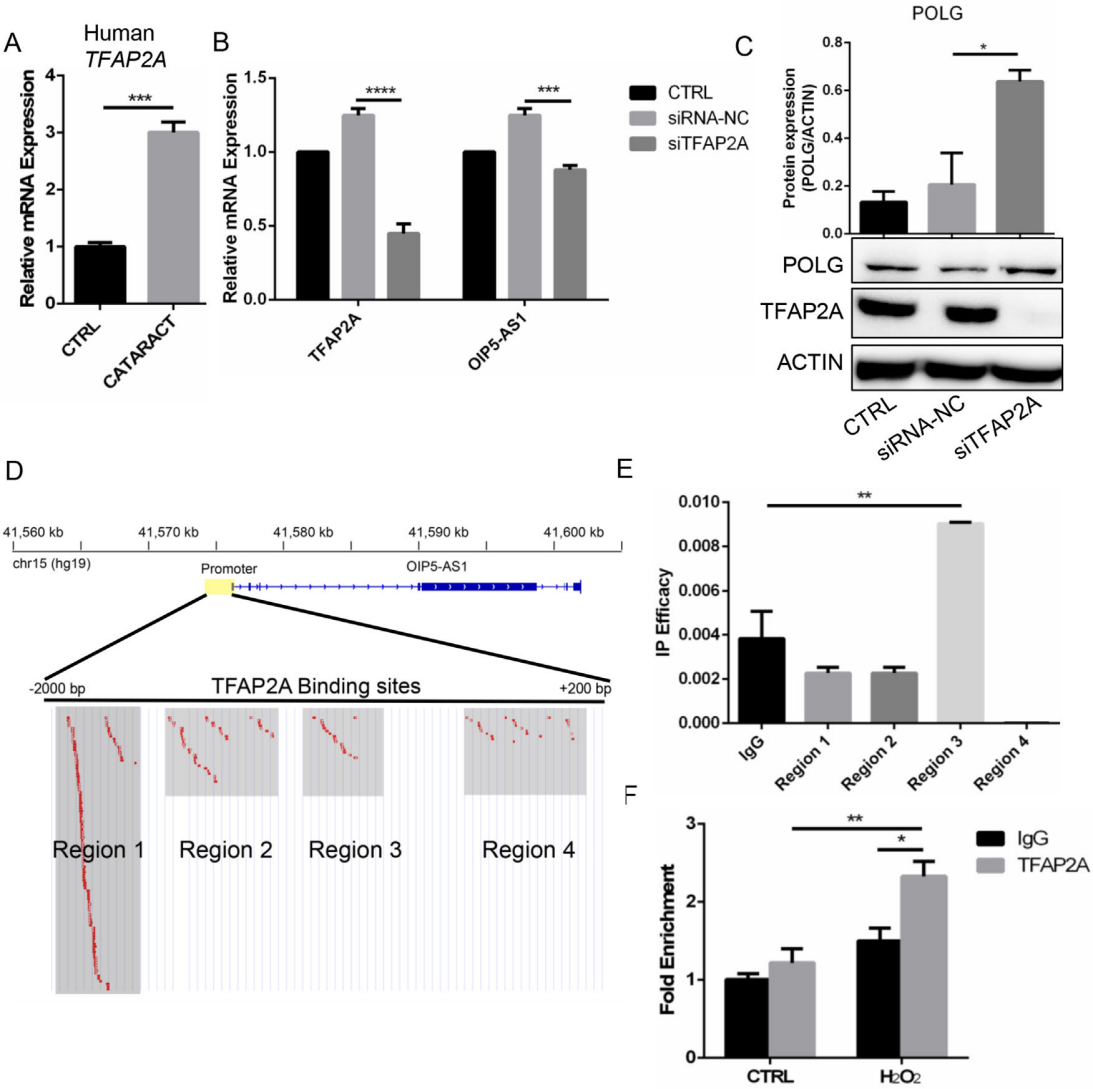
Prediction of *OIP5-AS1* transcriptional activation. (**A**) TFAP2A expression in the anterior capsule of patients with cataracts was higher than that of healthy individuals. (**B**) *OIP5-AS1* expression after TFAP2A knockdown. (**C**) *POLG* protein expression after OIP5-AS1 knockdown. (**D**) Prediction of *OIP5-AS1*–binding transcription factors identified TFAP2A. (**E**) IP efficacy of four regions around the *OIP5-AS1* transcription start site manually divided according to the distribution. (**F**) ChIP-qPCR of TFAP2A after 6 hours of 50 µM H_2_O_2_ induction. Primers were located in region 3. *P < 0.05, **P < 0.01, ***P < 0.001, ****P < 0.0001.

We demonstrated the important roles of *OIP5-AS1* in cataract formation. To further explore potential upstream transcription factors of *OIP5-AS1*, we conducted a motif analysis in the region surrounding the promoter of *OIP5-AS1*. We found that the transcription factor TFAP2A had the most predicted binding sites (Supplementary Table S2). TFAP2A expression of the anterior capsule of patients with cataracts was significantly higher than that in healthy subjects (*P* < 0.001, Fig. 7A) in data from Wu et al,^23^ indicating the potential regulatory role of TFAP2A in cataracts. To validate the regulatory effect of TFAP2A on *OIP5-AS1*, we silenced *TFAP2A* in B3 cells and detected significantly decreased expression of *OIP5-AS1* (*P* < 0.001, Fig. 7B) and elevated *POLG* expression (*P* < 0.05, Fig. 7C). According to our prediction, the TFAP2A binding sites in the *OIP5-AS1* promoter could be divided into four clustered regions (Fig. 7D). qPCR revealed that region 3 had the highest binding affinity compared with that of IgG (*P* < 0.01, Fig. 7E). Region 3 was the nearest region to the transcriptional start site of *OIP5-AS1*, further supporting the potential key role of TFAP2A in regulating *OIP5-AS1* transcription. We further performed ChIP-qPCR in the H_2_O_2_-induced B3 cells and observed significant enrichment in the TFAP2A treatment group compared with the negative control IgG group (*P* < 0.05, Fig. 7F). Altogether, these results indicated that TFAP2A might be involved in the upstream transcriptional regulation of *OIP5-AS1* in B3 cells under oxidative stress.

## Discussion

Apoptosis is a common cell feature of the pathogenesis of age-related cataract, and multiple factors and genes have been confirmed to be involved in the occurrence or development of this process.^35^ Herein, we proposed (Supplementary Fig. S3) that lncRNA *OIP5-AS1* was strongly transcriptionally activated in B3 cells from senile cataracts. Highly expressed *OIP5-AS1* and *POLG* mRNA were bound and delivered by the HuR protein to stress granules, resulting in *POLG* mRNA decay and ROS production. These changes led to endogenous apoptotic pathway activation and finally cell death. Moreover, we found that TFAP2A might be the upstream transcription factor regulating *OIP5-AS1* expression. We elucidated the specific functions of *OIP5-AS1* and *POLG*, investigated the upstream regulators, and expanded our knowledge of the lncRNAs involved in ARCs. Thus, we demonstrated a potential mechanistic basis for the association between *OIP5-AS1* and cataracts, which highlights the regulatory effects of lncRNAs on the pathogenesis of age-related ophthalmologic disease. More functional lncRNAs need to be identified and investigated. Despite the limitations, interfering with lncRNAs might be a novel strategy to prevent cataracts in the future.

Mutations in mtDNA have been found in many aging cells or tissues.^36^ Both depletion and deletion of mtDNA resulting from *POLG* alterations were associated with a group of heterogeneous diseases.^37^ The accumulation of ROS generated from oxidative phosphorylation damaged the mitochondrial membrane, causing the MMP to decrease and leading to the initiation of apoptosis.^38^ Lymphocytes with *POLG* mutations are more sensitive to oxidative stress-induced apoptosis than wild-type cells.^39^ In our study, *POLG* depletion led to a decrease in the mtDNA copy number and a decrease in MMP. With H_2_O_2_ stress, ROS production and proapoptotic protein expression increased, whereas *POLG* overexpression reversed these effects. In *POLG* knockdown experiments, H_2_O_2_ did not induce significantly more ROS production, apoptotic cells, or proapoptotic protein expression after *POLG* silencing, which was perhaps due to the severe oxidative damage induced by *POLG* depletion. In the *POLG* overexpression analyses, vector transfection created excessive oxidative damage; thus, H_2_O_2_ could not induce more oxidative stress. Additionally, we performed *OIP5-AS1* and *POLG* double knockdown and analyzed the ROS, MMP, mtDNA copy number, and apoptotic proteins to study the effects of the knockdown. The results indicated that the double silencing of *OIP5-AS1* and *POLG* had no significant effects on the cell fate and metabolic balance because *POLG* siRNA prevented the increases induced by *OIP5-AS1* silencing (Supplementary Fig. S2). Thus, *POLG* regulated mitochondrial function and cell fate.

HuR (formally known as ELAVL1) is a well-known RNA binding protein with broad function in diverse cellular activities.^40^ Generally, HuR was found to stabilize mRNA, resulting in enhanced protein translation.^41^^,^^42^ HuR was also found to function with AUF1, a cofactor, to promote p16 mRNA decay.^34^ Additionally, HuR could repress translation of p27, an important cell growth inhibitor.^43^ The lncRNAs often act as molecular sponges or decoys for RNA-binding proteins.^32^^,^^44^^,^^45^ Notably, *OIP5-AS1* is an AT-rich (including 29 continuous A) lncRNA, indicating that it could be bound by RNA binding protein.^46^ A previous study suggested that high level *OIP5-AS1* could prohibit the interaction of HuR with other mRNA by being a sponge increasing *OIP5-AS1*-HuR complex interaction.^32^ In our study, HuR not only bound *OIP5-AS1* mRNA, but also *POLG* mRNA, and *POLG* mRNA finally decreased. Therefore, we concluded that HuR might act as a scaffold delivering *OIP5-AS1* and *POLG* mRNA to the stress granules, in which *POLG* mRNA was destabilized or partially decayed. Intriguingly, *HuR* mRNA expression was enhanced by *OIP5-AS1* knockdown. Indeed, a positive feedback loop might be formed.^47^ In sum, our findings are consistent with HuR's role in controlling cell senescence.

Our study indicated that excess *OIP5-AS1* was expressed in lens epithelial cells from individuals with cataract. However, the reason for *OIP5-AS1* overexpression or downregulation in tumors or other tissues is unknown. To address this issue, we predicted transcription factors by bioinformatics methods. TFAP2A was identified, and further experiments verified that knockdown of TFAP2A decreased *OIP5-AS1* expression and that TFAP2A could bind to the *OIP5-AS1* promoter region near the transcription start site, which is consistent with previous findings.^48^ However, silencing TFAP2A only induced a slight downregulation of *OIP5-AS1*, suggesting that other transcription factors may be involved. Indeed, in the human genome, the majority of genes are regulated by multiple promoters and display tissue-specific regulation patterns.^49^ Further investigations are required to elucidate the precise mechanisms based on specific analyses, such as DNA pulldown-mass spectrometry.

TFAP2A was shown to regulate *OIP5-AS1* expression according to our predictions and experiments. Usually, TFAP2A is only expressed in lens epithelial cells, but not fiber cells.^50^ *TFAP2A* mutations were found to be associated with lens morphologic defects^50^ or cataracts with branchio-oculo-facial syndrome,^51^ suggesting the essential function of this molecule in the development of cataracts. Specifically, TFAP2A has cell-autonomous regulatory effects on lens vesicle separation. Cell adhesion and epithelial cell phenotype maintenance associated genes, such as E-cadherin, were regulated by TFAP2A. Upregulated expression of TFAP2A in fiber cells resulted in microphthalmia and cataracts in transgenic mice but blocked fiber cell differentiation.^52^^,^^53^ Moreover, TFAP2A was involved in the original differentiation and development of the neural crest as a pioneer transcription factor, indicating that TFAP2A could constitutively bind nucleosomal target sites and reorganize local chromatin to increase its accessibility.^54^^,^^55^ *TFAP2A* should be further investigated in both congenital and age-related cataracts.

We also analyzed *TFAP2A/OIP5-AS1/POLG* expression in human normal corneas based on the work of Kabza et al^56^ and retinas (GSE102485). Specifically, *OIP5-AS1* expression is higher than that of *TFAP2A* and *POLG* in the cornea and retina (Supplementary Fig. S4A, B). We found that *OIP5-AS1* could negatively regulate *POLG* expression in human corneal epithelial (HCE) cells and *TFAP2A* could positively regulate *POLG* expression in RPE (retinal pigment epithelium) cells (Supplementary Fig. S4D, G). However, we did not observe similar transcription activation of TFAP2A on *OIP5-AS1* in HCE (Supplementary Fig. S4C) and *OIP5-AS1* does not seem to be involved in TFAP2A/POLG regulation in RPE (Supplementary Fig. S4G, H). In addition, we have not detected the apoptosis-inducible effects of *OIP5-AS1* in HCE or RPE cells (Supplementary Fig. S4E, F, I, J), suggesting the lens-specific role of *OIP5-AS1*. Collectively, these experiments indicated that the whole *TFAP2A/OIP5-AS1/POLG* regulatory axis was only partially demonstrated in HCE/RPE cells, indicating the lens-specific regulatory mechanisms.

In summary, loss-of-function and gain-of-function experiments as well as interaction analyses elucidated a potential mechanistic basis for the relationship of *OIP5-AS1* to its target gene, *POLG*. Herein, we used immortalized cell line B3 to investigate the specific molecular mechanisms underlying *TFAP2A/OIP5-AS1/POLG* axis in cataract pathopoeias in vitro. The immortalized cell line likely could not fully adequately represent the primary lens cell line or lens in vivo. Further confirmation in primary cells or *OIP5-AS1* knockout animals is required.

## Supplementary Material

Supplement 1

Supplement 2

Supplement 3
