# Supplementary material for: Long Noncoding RNA OIP5-AS1 Inhibits Cell Apoptosis and Cataract Formation by Blocking POLG Expression Under Oxidative Stress
Source: Invest Ophthalmol Vis Sci. 2020 Oct 2;61(12):3. doi: 10.1167/iovs.61.12.3 (PMC7545078; doi:10.1167/iovs.61.12.3)
Supplement: Supplement 1 [file iovs-61-12-3_s001.docx]

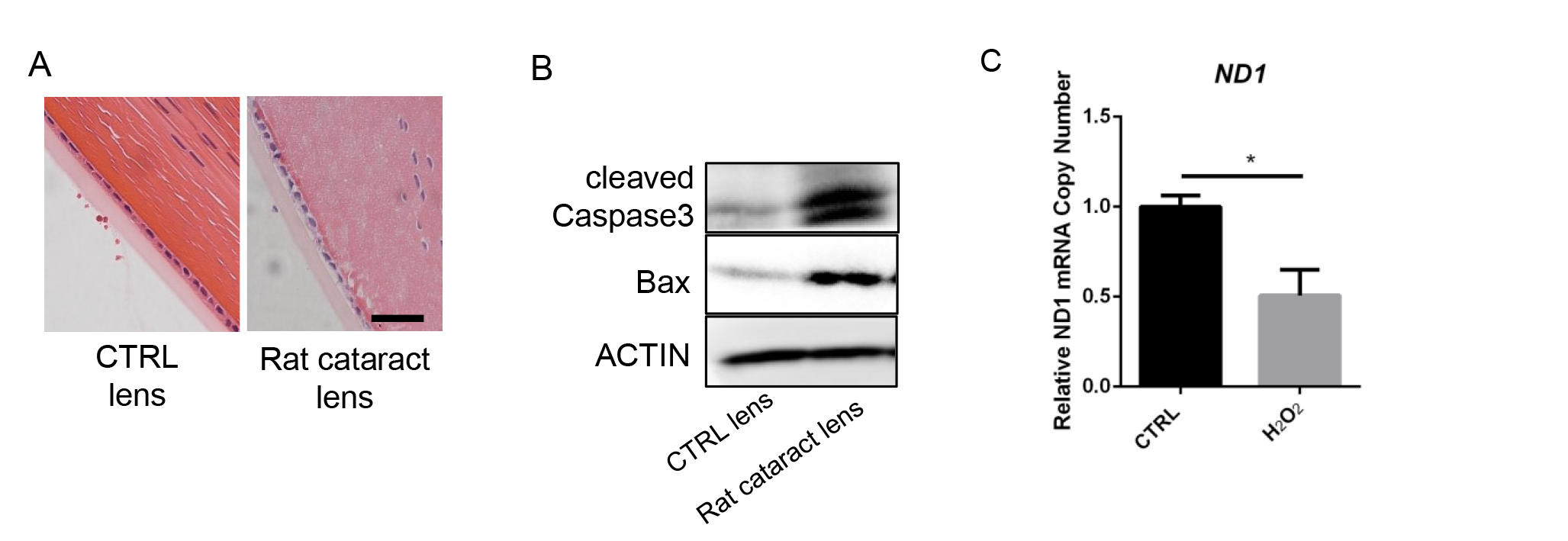


**Figure S1: Structure and specific index analysis of cataract models.**

A：H&E stain of rat lens of cataract model. B: Western blot detection of rat lens. C: MtDNA copy number of oxidative stress cell model. Scale bar: 50 μm.


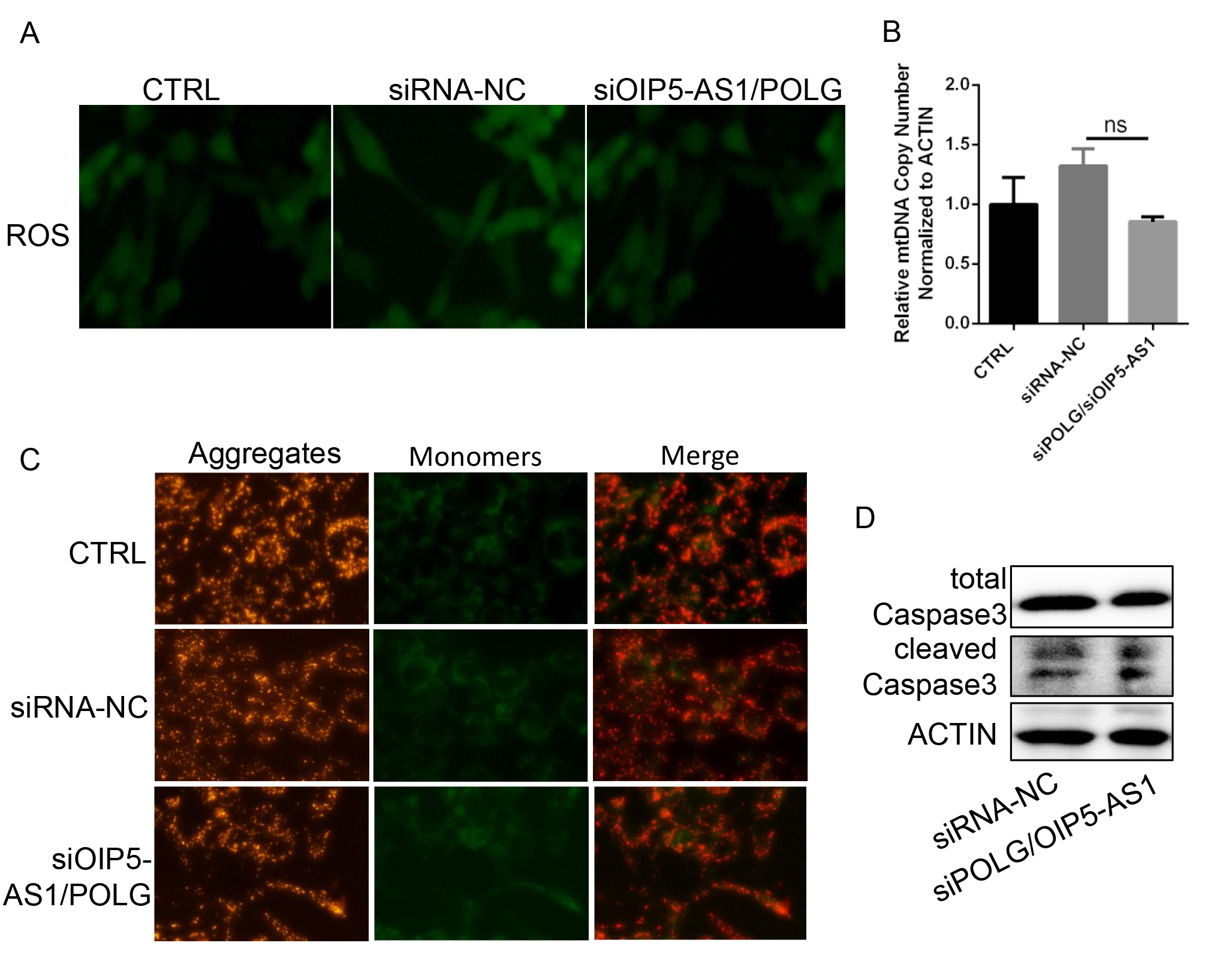


**Figure S2 Efffects of *OIP5-AS1/POLG* double knockdown.**

A: ROS images of *OIP5-AS1/POLG* silence. B: MtDNA copy number of *OIP5-AS1/POLG* silence. C: JC-1 stain of *OIP5-AS1/POLG* silence. D：Caspase3 expression after *OIP5-AS1/POLG* silence.


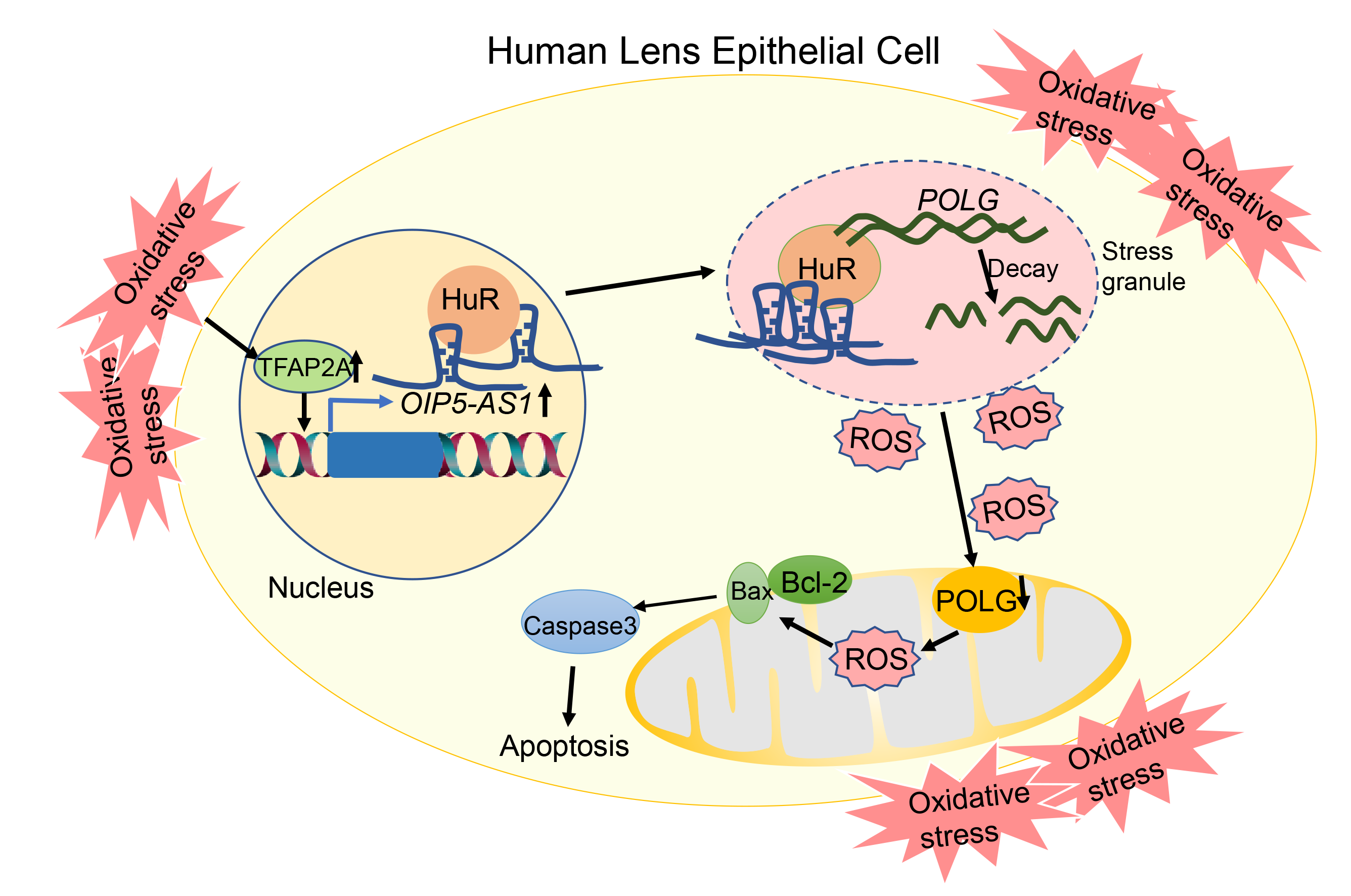


**Figure S3 schematic diagram of this study.**

TFAP2A, activated by oxidative stress in senile HLECs, promoted *OIP5-AS1* transcription. *OIP5-AS1* contributed to ROS production via promoting *POLG* mRNA decay mediated by HuR in stress granule, leading to apoptosis of HLECs.


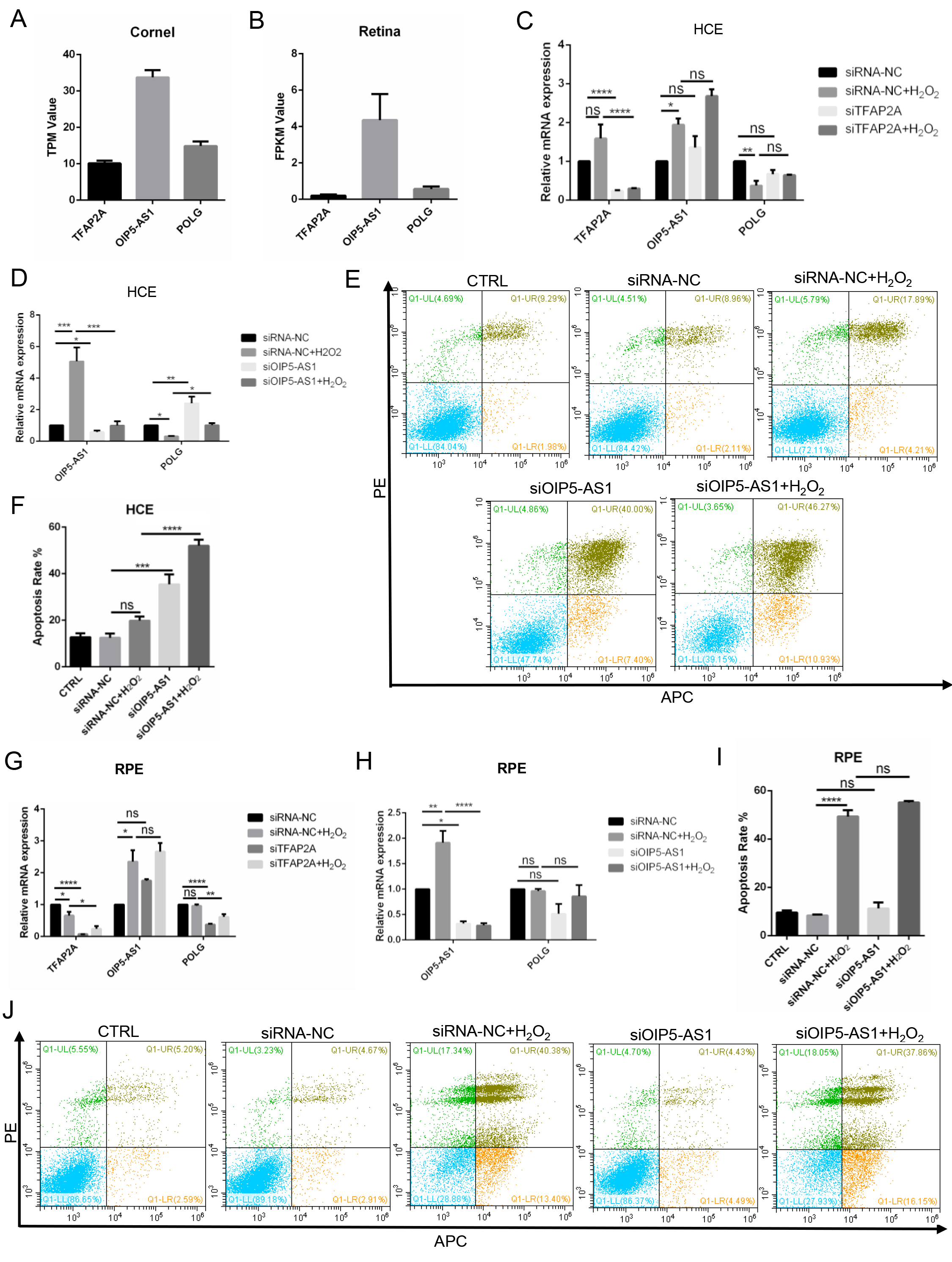


**Figure S4 Role of *TFAP2A, OIP5-AS1* and *POLG* in cornel and retina.** A: Expression value of *TFAP2A*, *OIP5-AS1* and *POLG* in human normal cornel sample (TPM, n=8). B: Expression of TFAP2A, OIP5-AS1 and POLG in human normal retina samples (FPKM, n=3). C-D: RT-qPCR examination in HCE of *OIP5-AS1* and *POLG* after *TFAP2A* and *OIP5-AS1* knockdown respectively. E-F: Flow cytometry of HCE apoptosis after *OIP5-AS1* knockdown with or without H_2_O_2_ and data was statistically analyzed in F. G-H: RT-qPCR examination of *OIP5-AS1* and *POLG* in RPE after *OIP5-AS1* and *TFAP2A* knockdown respectively. I-J: Flow cytometry of RPE apoptosis after *OIP5-AS1* knockdown with or without H_2_O_2_ and data was statistically analyzed in I. Ns *p*>0.05, **p*<0.05, ***p*<0.01, ****p*<0.001, *****p*<0.0001.
